# Characterization of the complete chloroplast genome of *Abies chensiensis* (Pinaceae), an endemic to China

**DOI:** 10.1080/23802359.2018.1535851

**Published:** 2018-11-21

**Authors:** Yuemei Zhao, Tao Zhou, Xiaodan Chen, Xiao Zhang, Guoqing Bai, Guifang Zhao

**Affiliations:** a College of Biopharmaceutical and Food Engineering, Shangluo University, Shangluo, China;; b School of Pharmacy, Xi’an Jiaotong University, China Xi’an;; c Key Laboratory of Resource Biology and Biotechnology in Western China (Ministry of Education) College of Life Science, Northwest University, Shaanxi Province, China Xi’an;; d Shaanxi Engineering Research Centre for Conservation and Utilization of Botanical Resources, Xi’an Botanical Garden of Shaanxi Province, Xi’an, China

**Keywords:** *Abies chensiensis*, chloroplast, Illumina sequencing, phylogenetic analysis

## Abstract

*Abies chensiensis*, an endemic evergreen trees distributed in Qinling Mountains, is listed in China Species Red List as an endangered species. In this study, the complete chloroplast genome (cpDNA) sequence of *Abies chensiensis* was determined from Illumina pair-end sequencing data. The cpDNA is 121,329 bp in length, contains a large single copy region (LSC) of 72,843 bp and a small single copy region (SSC) of 46,126 bp, which were separated by a pair of inverted repeat (IR) regions of 1180 bp. The genome contains 120 genes, including 81 protein-coding genes, 4 ribosomal RNA genes, and 35 transfer RNA genes. The overall GC content of the whole genome is 38.3%, and the corresponding values of the LSC, SSC, and IR regions are 38.8, 37.1, and 37.5%, respectively. Phylogenetic analysis based on 20 chloroplast genomes indicates that *A. chensiensis* is closely related to *A. sibirica*.

Firs are important for the ecosystem because they are resistant to cold and drought (Liu 1971). among them, *Abies chensiensis* is an endangered plant that is usually found scattered in small forest fragments at elevations from 2300 to 3000 m in the Qinling-Daba mountain region of China (Shao and Xiang 2015). But, within the last few years, *A. chensiensis* has experienced unexplained mortality and it has been listed in The IUCN Red List (http://www.iucnredlist.org/details/42274/0). Research suggests that the observed mortality of *A. chensiensis* may be a result of human activity and gradual climate change (Jia et al. 2016). Therefore, it is urgently needed to conserve this species scientifically and effectively. Until now, Molecular genetic research of *A. chensiensis* is limited due to lack of genomic information (Shao and Xiang 2015; Wang et al. 2014; Zhan et al. 2014). Here, we assembled the complete chloroplast (cp) genome of *A. chensiensis* based on Illumina paired-end sequencing for phylogenetic studies and the protection of genetic resources.

The fresh leaves of a single individual of *A. chensiensis* were sampled from Shangluo (Shaanxi, China; 108°49′E, 33°37′N) and Voucher herbarium specimens were deposited at the Herbarium of Shangluo University. Genomic DNA was extracted from the fresh leaves using the modified CTAB method (Doyle 1987). Total DNA was used for the shotgun library construction and the subsequent high-throughput sequencing on the Illumina HiSeq 2500 Sequencing System. In total, 3.9G raw reads were obtained, quality-trimmed and assembled using MITObim v1.8 (Hahn et al. 2013) with the reference sequence of *Abies sibirica* (GenBank: NC_035067). The genome was annotated using software Geneious v 9.0.2 (Biomatters Ltd., Auckland, New Zealand) by aligning with the reference chloroplast genome.The circular plastid genome map was completed using the online program OGDRAW (Lohse et al. 2013). The annotated chloroplast genome sequence has been deposited into the GenBank with the accession number MH796673.

The whole genome of *A. chensiensis* was 121,329 bp in length and contained two very short inverted repeat (IRa and IRb) regions of 1180 bp, which was separated by a large single-copy (LSC) region of 72,843 bp and a small single-copy (SSC) region of 46,126 bp. The cpDNA of *A. chensiensis* comprised 120 genes, including 81 protein-coding genes (74 PCG species), 4 ribosomal RNA genes, and 35 transfer RNA genes. In these genes,12 genes contained one intron, and two genes contained two introns. Most of the genes occurred as a single copy, while three tRNA gene species were duplicated. The overall GC content of *A. chensiensis* chloroplast genome is 38.3% and the corresponding values in LSC, SSC, and IR regions are 38.8, 37.1, and 37.5%, respectively.

The neighbor-joining(NJ) phylogenetic tree was constructed with MEGA 6.0 program (Tamura et al. 2013) based on 19 complete chloroplast genome sequences of Pinaceae and *Juniperus bermudiana* (Cupressaceae)(NC_024021) as an outgroup (Figure 1). The results indicated that all species in *Abies* clustered together and formed a monophyletic clade with *Keteleeria davidiana, Nothotsuga longibracteata, Pseudolarix amabilis* and *Cedrus deodara.* The tree also showed a close relationship between *A. chensiensis* and *A. sibirica.* In conclusion, this complete chloroplast genome would establish a solid foundation for future conservation genetic studies of *A. chensiensis*.

**Figure 1.**
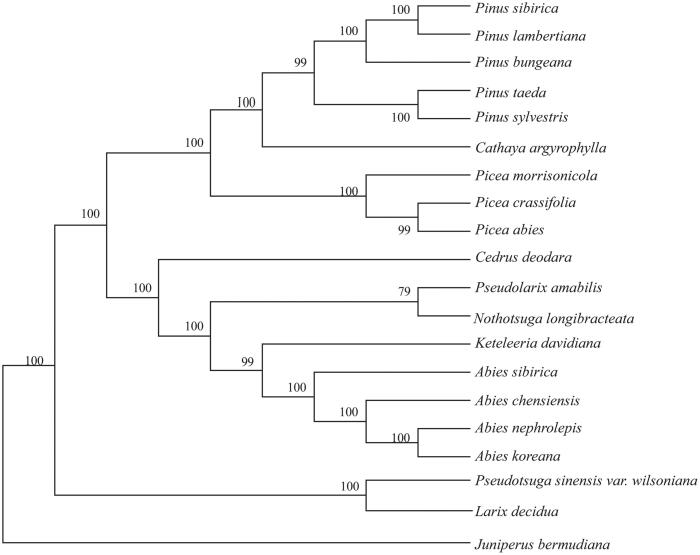
Neighbor-joining(NJ) phylogenetic tree based on 20 complete chloroplast genomes. Accession numbers: *Abies chensiensis* (MH_796673); *Abies sibirica* (NC_035067); *Abies nephrolepis* (KT_834974); *Abies koreana* (NC_026892); *Pseudolarix amabilis* (NC_030631); *Pseudotsuga sinensis var. wilsoniana* (NC_016064); *Pinus taeda* (NC_021440); *Pinus sylvestris* (NC_035069); *Pinus sibirica* (NC_028552); *Pinus lambertiana* (NC_011156); *Pinus bungeana* (NC_028421); *Picea morrisonicola* (NC_016069); *Picea crassifolia* (NC_032366); *Picea abies* (NC_021456); *Cathaya argyrophylla* (NC_014589); *Cedrus deodara* (NC_014575); *Keteleeria davidiana* (NC_011930); *Larix decidua* (NC_016058); *Nothotsuga longibracteata* (NC_037407); *Juniperus bermudiana* (NC_024021).
